# The function and mechanism of circular RNAs in gastrointestinal tumours

**DOI:** 10.1111/cpr.12815

**Published:** 2020-06-08

**Authors:** Hui Nie, Yutong Wang, Zhiming Liao, Jianhua Zhou, Chunlin Ou

**Affiliations:** ^1^ Department of Pathology Xiangya Hospital Central South University Changsha China; ^2^ Department of Pathology the Fourth Hospital of Changsha Changsha China

**Keywords:** circular RNAs, gastrointestinal tumours, inflammatory responses, treatment

## Abstract

Gastrointestinal tumours are tumours that originate in the digestive tract and have extremely high morbidity and mortality. The main categories include: oesophageal, gastric, and colorectal cancers. Circular RNAs are a new class of non‐coding RNAs with a covalent closed‐loop structure without a 5’ cap or a 3’ poly A tail, which can encode a small amount of polypeptide. Recent studies have shown that circRNAs are involved in multiple biological processes during the development of gastrointestinal tumours including proliferation, invasion and metastasis, radio‐ and chemoresistance, and inflammatory responses. Also, the clinical and pathological characteristics of the patient, such as staging and lymph node metastasis, are closely associated with the expression level of circRNAs. Further investigation of the function and the role of circRNAs in the development of gastrointestinal tumours will provide new directions for its clinical diagnosis and treatment.

## 1 INTRODUCTION

Gastrointestinal tumours are tumours that originate in the digestive tract and are associated with very high morbidity and mortality. The most common gastrointestinal tumours are oesophageal cancers (OC), gastric cancers (GC) and colorectal cancers (CRC).^1^ Among them, GC is one of the most critical malignant tumours worldwide. Though GC ranks fifth in cancer incidence, its mortality rate remains high and it ranks third in cancer‐related deaths.^2^ CRC ranks third in incidence among malignant tumours and fourth in cancer‐related deaths.^3^ OC ranks eighth and sixth in morbidity and mortality, respectively. Although the morbidity of OC is lower compared with CRC and GC, it is still one of the common malignant tumours worldwide.^4^ With the improvements in treatment and living standards, the survival time of patients with early‐stage tumours of the digestive system has been extended significantly, but the five‐year survival rate for patients with advanced gastrointestinal tumours remains low. Therefore, finding early diagnosis markers and new therapeutic targets is an important strategy to improve the survival rate of patients with gastrointestinal cancers.

Circular RNAs (CircRNA) have a covalent closed‐loop structure without a 5’ cap and/or a 3’ poly A tail.^5^ Based on whether they can be translated, circRNAs can be divided into non‐coding circRNAs and coding circRNAs.^6^ CircRNAs were first reported in RNA viruses by Sanger et al ^7^ in 1976. Subsequently, the presence of circRNAs was also confirmed in the cytoplasm of eukaryotes of many different species.^8^ In the beginning, circRNAs were considered as "junk" RNAs without any real function. They were regarded as by‐products of incorrect splicing, or by‐products of processing of precursor mRNAs at their low‐abundance stages.^9^ Until 2012, large numbers of circRNAs were discovered and identified owing to the advancements in high‐throughput sequencing.^8^ As research progressed, it became increasingly clear that circRNAs play an important role in various cellular activities and development.^10^ Existing evidence shows that circRNAs are closely associated with several pathological and physiological processes in tumours including growth, differentiation, metastasis and invasion of cancer cells.^11^ Liu et al ^12^ demonstrated that circRNA YAP1 inhibits proliferation and invasion of gastric cancer cells. circITGA7 has been found that it has the ability of promoted the growth and metastasis of colorectal cancer cells by Li et al^13^ Xia et al ^14^ observed that circ_0067934 promotes the differentiation of OC cells. A growing number of studies have characterized circRNAs as early diagnostic and prognostic markers. Beyond that, circRNAs can also serve as potential therapeutic targets.^15^, ^16^ Although several studies have focused on the circRNAs and tumours of the digestive system, the precise roles and mechanisms of circRNAs remain unclear. Therefore, further elucidation of the specific roles and mechanisms of circRNAs in the development of digestive system tumours is of great significance for guiding clinical diagnosis and treatment.

## 2 THE FUNCTION OF CIRCRNA

### 2.1 Biological Characteristics of CircRNAs

**Figure 1:**
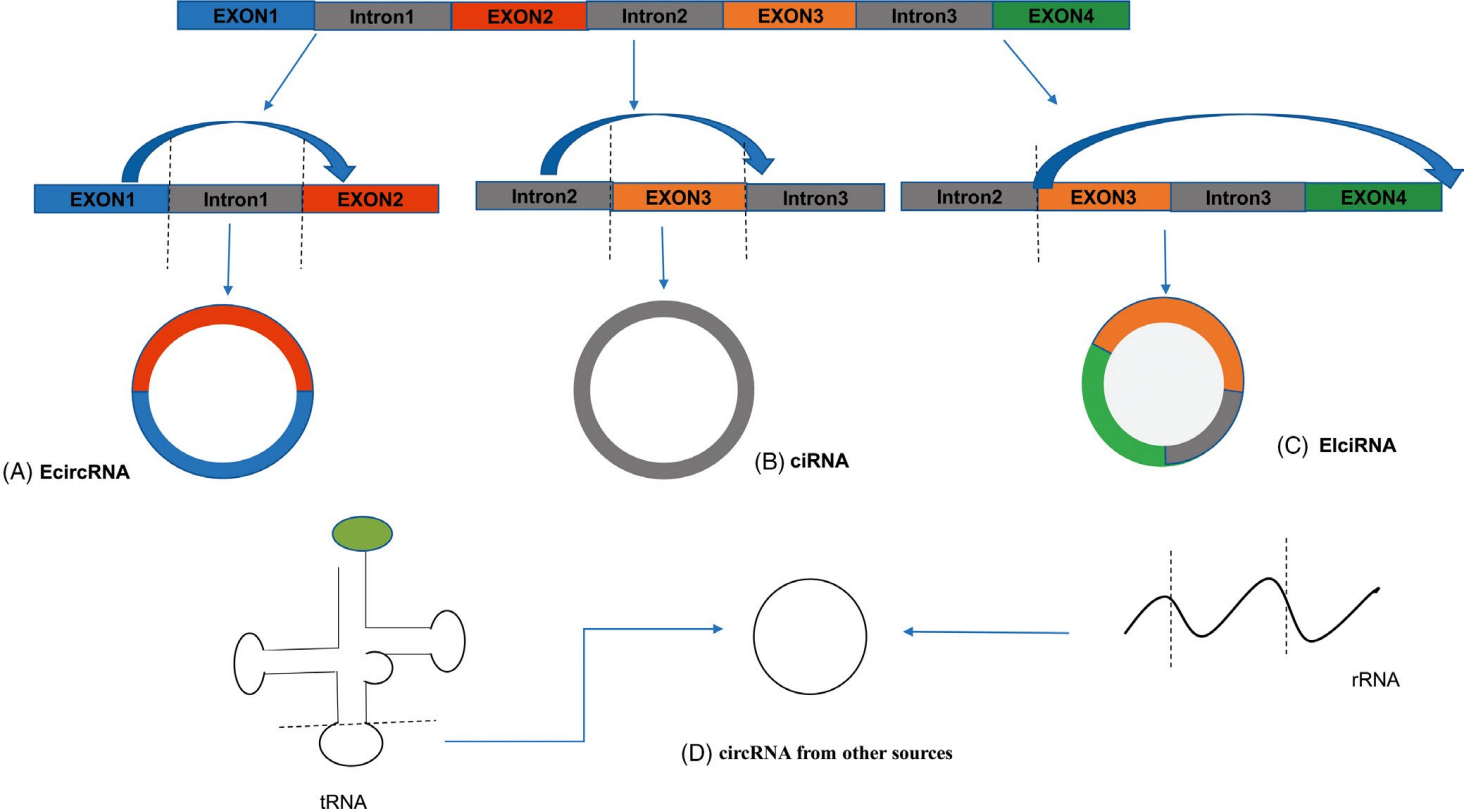
The sources circRNAs formation. (A) Exonic circRNAs (EcircRNAs): Most circRNAs are formed in this way, and these circRNAs are exclusively formed by cyclization of exons without flanking introns. (B) Intron‐derived circRNAs (ciRNAs): the ciRNAs are formed by the cyclization of introns and are composed of one or more introns and are mainly expressed in the nucleus. (C) Retained‐intron circRNAs (EIciRNA): the EIciRNA are derived from the exons and introns of pre‐mRNAs and are also known as reverse transcription of intron reservation. These circRNAs are mainly expressed in the nucleus. (D) Other sources of circRNA: In addition to the above three main sources, circRNAs can also be generated by cyclization of viral RNA genomes, tRNA, rRNA and snRNA among others. The formation of circRNAs is relatively complex process involving a series of biological steps

CircRNAs are a newly discovered class of endogenous ncRNAs. Unlike conventional linear RNAs, the 3’ and 5’ ends of circRNAs are ligated to form a covalent closed‐loop structure.^3^ CircRNAs are mainly composed of exons and/or introns.^17^ According to their source of sequence, circRNAs can be classified into four categories (Figure 1), namely：1）Exonic circRNAs (EcircRNAs), composed of exons only and found mainly in the cytoplasm; 2）Intron‐derived circRNAs (CiRNAs), composed of introns and mostly expressed in the nucleus; 3）Retained‐intron circRNAs (EIciRNAs), composed of exons and introns and mainly expressed in the nucleus^9^; and 4) Virus circRNAs, generated by circularization of viral RNA genomes, tRNAs, rRNAs and snRNAs among others.^18^, ^19^ According to the different ways of cyclization, circRNAs can be divided into three types: Spliceosome‐dependent cable tail patching circRNAs formation, cis‐acting elements promoted circRNAs formation and RNA‐binding protein regulated circRNAs formation.^20^ Most studies have shown that circRNAs are highly conserved and stabile, and abundant. In addition, circRNAs are expressed at different levels in different tissues and cells, which means that they also tissue‐ and cell‐specific.^21^

### 2.2 Function and Method of CircRNAs

As an emerging class of ncRNAs that regulate gene expression, the functional mechanisms of circRNAs have received widespread attention. Several recent studies have shown that circRNAs exert their effects mainly in the following ways: 1) Because they contain miRNA‐binding sites, circRNAs can act as miRNA sponges (Table 1). They can indirectly regulate the expression of miRNA downstream target genes by preventing the miRNAs from binding to the 3’ untranslated regions of the mRNAs. 2) CircRNAs, along with RNA‐binding proteins (RBPs), play an important role in changing the RNA splicing modes and mRNA stability. Du et al found that circ‐foxo3 binds with CDK2 and P21 to form an RNA‐protein complex to inhibit cell cycle progression from G1 to S phase.^22^ In addition, circRNAs also interact with RNA polymerases to affect the process of gene transcription. Furthermore, the interactions between ElcircRNAs, microribonucleoprotein and RNA polymerase have an important effect on gene transcription in vivo. For example, EiciRNAs—u1 SNRNP compounds, which are formed by circ‐EIF3J and circ‐PIAP2 can interact with RNA polymerase Ⅱ to promote the process of parental gene transcription.^23^ 3）While it is well known that circRNAs are ncRNAs without 5’ caps or 3’ poly A tails, some studies have found that many circRNAs have internal ribosome entry sites or open reading frames that participate in the transcription and translation of functional proteins. For example, circ‐ZNF609 has two initiation factors, the existence of which makes it possible to encode functional proteins.^24^ Zheng et al ^25^ revealed that some open reading boxes with translational function exist in circPPP1R12A and the proteins encoded by circPPP1R12A may play an vital role in the formation and progression of CRC. The results of a recent study showed that circ‐PINT, formed by the individual exon cyclization of lncRNA LINE‐PINT, has a highly conserved open reading frame that can be translated by internal ribosome entry sites (IRES), resulting in the creation of a completely new peptide consisting of 87 amino acids.^26^ 4) Besides these functions, circRNAs can also act as “miRNA reservoirs” which can release large amounts of miRNAs in certain circumstances to inhibit the expression of target genes. The role of circRNAs in releasing miRNAs was first identified during the study of the ciRS‐7, miR‐7 and miR‐671 regulatory networks. Hansen et al^27^ demonstrated that the highly expressed ciRS‐7 has the ability of storing miR‐7s and releasing them in specific places and at specific times to suppress miR‐671‐stimulated expression of miR‐7. Although the existing reports on circRNAs have laid the foundation for understanding its important cellular roles, it is still necessary to verify the functions that remain unclear and further explore the hereto unknown mechanisms of action of circRNAs.

**Table 1:** Overview of the circRNAs‐miRNAs‐target genes/pathway in gastrointestinal tumours

	circRNAs	miRNA sponge	Target gene/pathway	Dyregulation	Functions (+) represents promotion, (‐)represented inhibition	References
OC	circ_0006168	miR‐100	mTOR	Up	Proliferation(+),migration(+),invasion(+)	^4^
	Circular RNA ciRS‐7 circ‐PRKCI circ_0004370	miR‐7 miR‐3680‐3p miR‐1294	KLF4 AKT3 LASP1	Up Up Up	migration(+),invasion(+) Proliferation(+),migration(+), Proliferation(+),invasion(+)	^56^ ^98^ ^77^
GC	circ_0006848 circHECTD1 circRNA YAP1 circ_0000592 circCACTIN circ_0008035 circ AKT3 circ PSMC3 circNRIP1 circ‐SFMBT2 circ‐0027599 circHIPK3 circLARP4 circRNA‐100269 circOSBPL10 circ‐SPECC1 circ_006100 circ‐NOTCH1 circ‐SERPINE2 circ‐0001368 circ‐0067997 circRNA NF1 circFAT1(e2) circPDSS1 circRNA 001 569 circRNA CDR1as circ_0081143 circ_0000673 circ‐ZFR circ‐RanGAP1	miR‐329‐5p miR‐1256 miR‐367‐5P various miR‐331‐3p miR‐375 miR‐198 miR‐296‐5P miR‐149‐5p miR‐182‐5p miR‐101‐3p.1 miR‐124/miR‐29b miR‐424 −5p miR‐630 miR‐136‐5p miR‐526b miR‐195 miR‐637 miR‐375 miR‐6506‐5P miR‐515‐5P miR‐16 miR‐548g miR‐186‐5P miR‐145 miR‐7‐5p miR‐646 miR‐532‐5p miR‐130a/miR‐107 miR‐877‐3p	RPL6 USP5 p27 Kip1 various TGFBR1 YBX1 PIK3R1 PTEN ATK1 CREBI PHLDA1 various LATS1 LPHN2 WNT2 KDM4A/YAP1 GPRC5A Apelin YWHAZ FOXO3 XIAP MAP7/AKT3 RUNX1/YBX1 NEK2 NR4A2 REGγ CDK6 RUNX3 ZFR/PTEN VEGFA	Down Up Down Up Up Up Up Down Up Up Down Up Down Down Up Down Up Up Up Down Up Up Down Up Up Down Up Down Down Up	—— Proliferation(+),migration(+),invasion(+) Proliferation(‐),invasion(‐) Proliferation(+),migration(+) Proliferation(+),migration(+),invasion(+),EMT Proliferation(+),invasion(+) Cisplatin resistance (+) Proliferation(‐),migration(‐) Proliferation(+),migration(+),invasion(+) Proliferation (+) Proliferation(‐),migration(‐) —— Proliferation(‐),invasion(‐) Proliferation (‐) Proliferation(‐),migration(‐),invasion(‐) Proliferation(‐),invasion(‐) Proliferation(+),migration(+),invasion(‐) Proliferation(+),invasion(+) Proliferation (+) Proliferation (‐),invasion(‐) Cell viability (+),Colony formation （+） Proliferation (+) Proliferation(‐),migration(‐),invasion(‐) Cancer progression (+) Proliferation(+),Cell viability (+) Toxicity of DB (+) Cells viability(+),invasion（+）,sensitivity cisplatin (+) Proliferation(‐),invasion(‐) Proliferation(‐),cell cycle(‐) Migration(+),invasion(+)	^84^ ^80^ ^12^ ^41^ ^57^ ^99^ ^67^ ^45^ ^100^ ^43^ ^46^ ^101^ ^86^ ^48^ ^102^ ^103^ ^104^ ^105^ ^106^ ^107^ ^108^ ^109^ ^110^ ^111^ ^112^ ^68^ ^113^ ^114^ ^47^ ^115^
CRC	circ_0000523 circRNA CBL.11 circ HIPK3 circRNA 100 290 circ ITGA7 circ ITGA7 circRNA ciRS‐7‐A circ000984 circ_102958 circ_001569 circ_0020397 circ_0136666 circ_103809 circ ‐ACAP2 circ_0026344 circ‐CCDC66 circ_0000218 circ_0021977 circ_0007142 circ_0009361 circVAPA circIFT80 circ_0053277 circ_0079993 circ101555 circ‐NSD2 Circ‐ZNF609 circ_0071589	miR‐31 miR‐6778‐5P miR‐7 miR‐516b miR‐370‐3P miR‐3187‐3p miR‐7 miR‐106b miR‐585 miR‐145 miR‐138 miR‐136 miR‐532‐3p miR‐21‐5P miR‐183 miR‐1238‐3p miR‐139‐3p miR‐10b‐5p miR‐103a‐2‐5p miR‐582 miR‐101 miR‐1236‐3p miR‐2467‐3p miR‐203a‐3p.1 miR‐597‐5p miR‐199b‐5p miR‐150 miR‐600	Wnt/β‐catenin YWHAE various FZD4/ Wnt/β‐catenin ITGA7 ASXL1 EGFR/RAF1 CDK6 CDC25B E2F5/BAG4/FMN2 PD‐L1/TERT SH2B1 FOXO4 Tiam1 CCL20/CXCL8 LHX2 RAB1A p21&p53 DOCK1 APC2 various HOXB7 MMP14 CREB1 CDK6/ RPA3 DDR1/JAG1 Gli1 EZH2	Down Down Up Up Down Down Up Up Up Up Up Up Down Up Down Up Up Down Up Down Up Up Up Up Up Up Up Up	Proliferation (‐) Proliferation (‐) Proliferation(+),migration(+) Proliferation(+),migration(+),invasion(+) Proliferation(‐),migration(‐) Proliferation (‐) Proliferation(+),migration(+),invasion(+) Proliferation(+),migration(+) Proliferation(+),migration(+),invasion(+) Proliferation(+),invasion(+) Cell viability(+),invasion(+) Proliferation(+),invasion(+) Proliferation(‐),migration(‐) Proliferation(+),migration(+),invasion(+) Migration (‐) Proliferation(+),migration(+) Proliferation(+),migration(+) Proliferation(‐),migration(‐),invasion(‐) Proliferation(+),migration(+) Proliferation(‐),migration(‐),invasion(‐),EMT(‐) Proliferation(+),migration(+),invasion(+) Proliferation (‐) Proliferation(+),migration(+),EMT Proliferation (+) Cancer progression (+) Migration(+) Migration(+) Proliferation(+),migration(+),invasion(+)	^3^ ^64^ ^52^ ^116^ ^13^ ^117^ ^88^ ^88^ ^118^ ^119^ ^53^ ^89^ ^120^ ^121^ ^122^ ^123^ ^124^ ^125^ ^126^ ^127^ ^128^ ^129^ ^130^ ^131^ ^132^ ^133^ ^134^ ^135^

## 3 CIRCRNAS AND GASTROINTESTINAL TUMOURS

**Figure 2:**
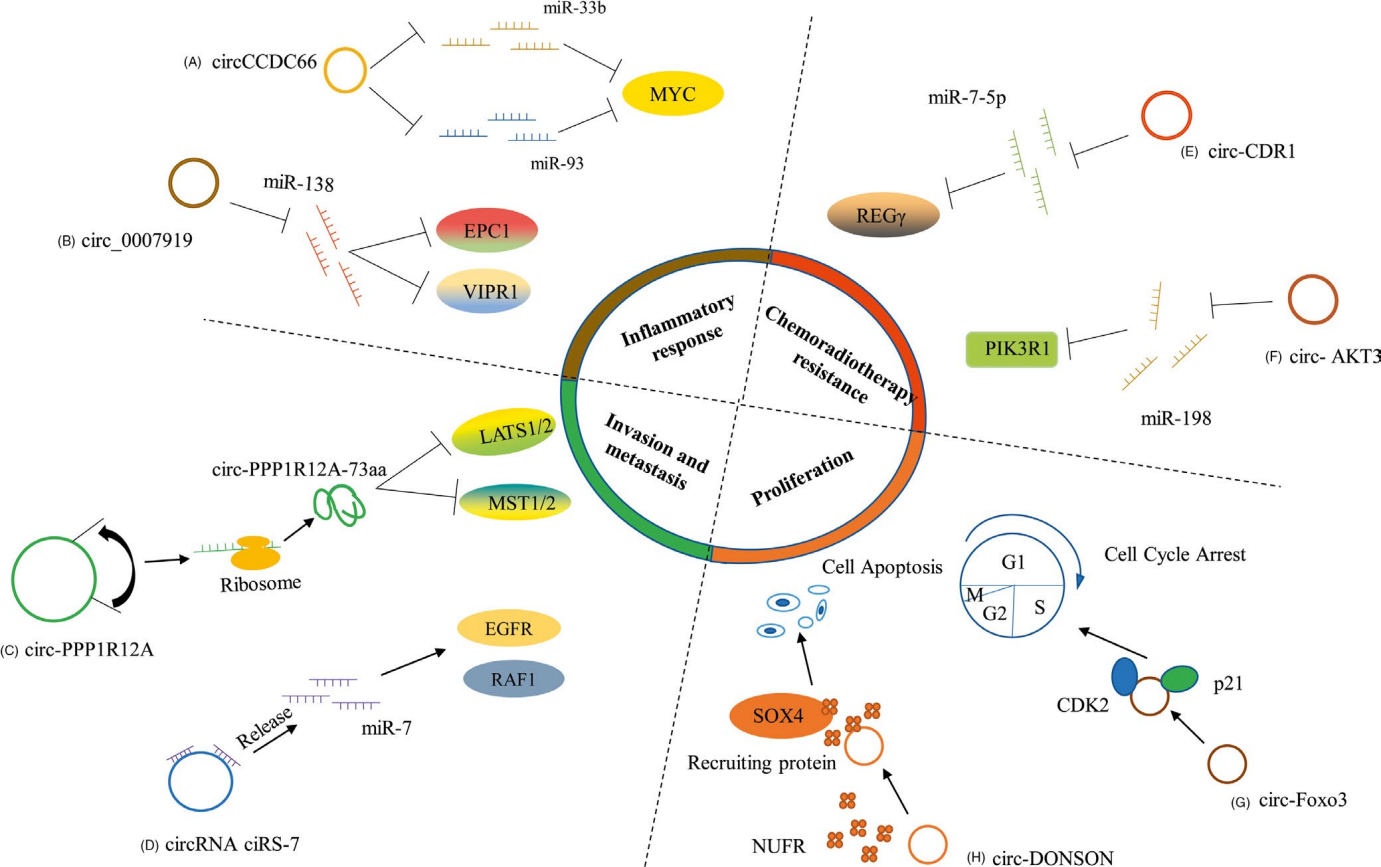
CircRNAs in Cancer Phenotypes. CircRNAs contribute to each of the hallmarks of cancer. Selected examples of circRNAs and their molecular partners or genomic targets are shown for proliferation, invasion and metastasis, inflammatory response and chemoradiotherapy resistance. (A) circ‐CCDC66 was associated with the occurrence and development of UC, and may be involved in a series of pathological processes of colonic polyps by regulating miR‐33b and miR‐93. (B) circ _0007919 may be involved in the course of UC by interacting with miR‐138 and let‐7a to regulate the potential target genes, EPC1 and VIPR1. (C) some open reading boxes with translational function exist in circPPP1R12A and the proteins encoded by circPPP1R12A may play an vital role in the formation and progression of CRC. (D) the overexpression of ciRS‐7 promoted the invasion and metastasis of OC cells through modulating miR‐7/KLF4 and NF‐ B signalling pathways. (E) circRNA CDR1 could cause REGγ overexpression in GC cells through sponging miR‐7‐5p. (F) By inhibiting miR‐198 and upregulating PIK3R1 gene expression, circRNA AKT3 promoted DNA damage and repair, inhibited apoptosis of GC cells, and increased their resistance to CDDP in vitro and in vivo. (G) circ‐foxo3 binds with CDK2 and P21 to form an RNA‐protein complex to inhibit cell cycle progression from G1 to S phase. (H) circ‐DONSON can induces apoptosis in GC cells by recruiting proteins

Tumorigenesis is the process of uncontrolled growth of cells which may be stimulated by internal and external carcinogens. The mechanism of tumorigenesis is complex and involves changes at the tissue, cellular and molecular levels. Hanahan and Weinberg^28^ reported that tumour cells possess several unique traits including self‐sufficiency of growth signals, insensitivity to antigrowth signals, evasion of apoptosis, limitless replicative potential, sustained angiogenesis, tissue invasion and metastasis, avoiding immune destruction, tumour promotion inflammation, deregulating cellular energetics and genome instability and mutation. Recent studies have shown that circRNAs play a significant role in the development of tumours, and are closely associated with the characteristics processes in tumours including proliferation, invasion and metastasis, chemoradiotherapy resistance and inflammatory response^29^ (Figure 2).

### 3.1 CircRNAs and the malignant proliferation of gastrointestinal tumours

The malignant proliferation of tumour cells involves multiple signalling pathways, such as the JAK‐STAT signalling pathway,^30^ which is involved in many important biological processes such as cell proliferation, differentiation, apoptosis and immune regulation; the NF‐kB signalling pathway,^31^ which inhibits apoptosis and is closely associated with the occurrence and development of tumour; the Ras, PI(3)K and mTOR signalling pathways ,^32^, ^33^ which have been studied extensively and are associated with various tumours; and the classical Wnt ^34^ and BMP signalling pathways ^35^ among others. For example, circ‐FBXW7 can mediate the proliferation, migration and invasion of CRC via activating the NEK2, mTOR and PTEN signalling pathways.^36^ Compared to normal cells in which proliferation is precisely regulated by growth factors and depends on the surrounding microenvironment, tumour cells can proliferate indefinitely. Several studies have demonstrated that tumour cells have the ability to produce large amounts of growth factors themselves to resist the dependence on external factors and disrupt the normal proliferation mechanism of cells in tissues to achieve infinite proliferative potential.^37^

According to some recent studies, circRNAs can promote the abnormal proliferation of the tumour cells in the digestive tract. Circ‐0007534, whose expression level is upregulated in CRC compared with normal intestinal tissues, is a typical example of one such circRNA. The silencing of circ‐0007534 inhibits the proliferation of CRC cells and induces apoptosis in tumour cells.^38^ Among the circRNAs involved in OC, Rong et al^39^ reported that the expression level of circ‐DLG1 was significantly upregulated in OC cells, tissues and plasma compared with normal oesophageal tissues. They also verified that knockout of circRNA inhibited the proliferation of OC cells. Song et al^40^ revealed that circ_0000337 mediates the progress of OC by sponging miR‐670‐5p. In another study, the expression level of circ‐0000592 was significantly increased in GC compared with normal tissues. The biological effects of circ_0000592, such as suppression of cell proliferation and cell cycle arrest at the G0/G1 phase, have been recognized as mediated through sponging of miRNAs.^41^ Studies have shown that circ‐DCAF6 can promote GC multiplication and growth by sponging miR‐1231 and miR‐1256.^42^ Sun et al found that^43^ circ‐SFMBT2 interacts with miR‐182‐5p and promotes the proliferation of GC cells by regulating the expression level of CREB1 mRNA. In addition, there are some circRNAs with lower expression in the cancer tissues compared to the adjacent normal tissues. For instance, circ_0000523 interacts with miR‐31 to restrain CRC cell growth by modulating the cancer‐related Wnt/β‐catenin signalling pathway.^3^ In colon cancer, Li et al^13^ revealed that circITGA7 represses the proliferation and metastasis of CRC cells by regulating the Ras signalling pathway, leading to the upregulation of ITGA7 gene expression. They also verified that the knockdown of circRNAs promotes the growth and metastasis of CRC cells in vitro. Further, low expression of circ‐ITCH in colon cancer represses the activation of MAPKs and alleviates the gene expression of ITCH thereby affecting the multiplication process.^44^ Compared to normal gastric tissue, circPSMC3 was expressed at lower levels in GC patients. In the tumour cells, circPSMC3 acts as a competitive endogenous RNA (ceRNA) to sponge miR‐296‐5p to regulate the expression of phosphatase and tensin homolog (PTEN), and further suppress the progression and development of gastric cancer. The low level of circPSMC3 may contribute to cell proliferation as well as cancer initiation.^45^ Wang et al ^46^ reported that, unlike in adjacent normal tissues, the expression of circ0027599 was significantly decreased in GC tissues. Functional studies showed that it affects the proliferation and metastasis of GC cells by sponging miR‐101‐3p. The expression of circ‐ZFR is significantly downregulated in GC, and it sponges miR‐130a and miR‐107 and affects the expression levels of ZFR and PTEN. Moreover, upregulation of circ‐ZFR inhibits the proliferation of GC cells and slows cell cycle progression.^47^ Studies suggest that circ100269 regulates the growth of tumour cells by sponging miR‐630 in GC.^48^ Although circRNAs have been reported to be widely involved in the proliferation of gastrointestinal tumour cells, the complex mechanisms involved in regulating the proliferation of tumour cells and their role in tumours remain to be explored.

### 3.2 CircRNAs and the invasion and metastasis of gastrointestinal tumour

The invasion and metastasis of tumour cells involves a series of pathophysiological changes, among which changes in adhesion of tumour cells to the surrounding cells and the extracellular matrix plays an important role. Epithelial‐mesenchymal transition (EMT) is also involved in tumour metastasis and invasion. EMT enables cancer cells to invade and metastasize, and cancer cells can activate EMT to varying degrees.^49^ Furthermore, growth factors such as EGF, IL‐1, TGF can stimulate the mobility of the tumour cells, which is critical in tumour invasion and metastasis.^50^

More and more reports have indicated that circRNAs are closely associated with the invasion and metastasis of gastrointestinal tumours. For example, circFNDC3B facilitates EMT and increases the expression of CD44, which is related to cell adhesion, by regulating the circFNDC3B‐IGF2BP3‐CD44 axis, thereby promoting invasion and metastasis of GC cells.^51^ Zheng et al ^25^ found that circPPP1R12A‐73aa, a functional protein encoded by circPPP1R12A, promotes the proliferation, migration, and invasion of GC. CircHIPK3 expression was found to be downregulated in CRC, and it was proposed to work through regulating the c‐myb/circHIPK3/miR‐7 axis. Besides, the silencing of circHIPK3 had a negative effect on CRC progression by impacting invasion, proliferation and migration.^52^ Zhang et al ^53^ reported that circ_0020397 enhances the vitality and invasiveness of CRC cells by binding with miR‐138. Circ_0020397 also inhibits apoptosis of cancer cells. Some studies reported that the expression levels of circRNA_0001178 and circRNA_0000826 were markedly upregulated in the hepatic metastasis of CRC patients compared to those without metastasis. To test whether there was a correlation between these two circRNAs and colorectal liver metastases, a circRNAs regulation network was constructed. The results revealed that the two circRNAs may have a key function in mediating liver metastasis in CRC.^54^ Similarly, Jiang et al ^55^ discovered several differentially expressed circRNAs between metastatic and non‐metastatic CRC cell lines (CRC cell line SW480 and CRC metastatic cell line SW620), which suggested that the differentially expressed circRNAs may play important role in CRC metastasis. In OC, the overexpression of ciRS‐7 promoted the invasion and metastasis of OC cells through modulating miR‐7/KLF4 and NF‐ B signalling pathways.^56^ In GC, circ‐CACTIN knockdown inhibits the proliferation, metastasis, invasion and EMT of GC cells. On the contrary, its overexpression promoted metastasis and invasion of GC cells without affecting proliferation. The possible biological mechanism may be through the regulation of related upstream and/or downstream genes such as miR‐331‐3p.^57^ In addition, some circRNAs are downregulated in gastrointestinal tumours. For example, downregulated expression of circ_0074362 was confirmed to inhibit the invasion of GC cells.^58^ Ding et al found ^59^ that the silencing of circ‐DONSON suppressed the proliferation, invasion and metastasis of GC and induced apoptosis of cancer cells. The expression of circ‐0000567 was decreased in CRC, and circ_0000567 knockout was found to promote tumour cell metastasis and proliferation.^60^ In addition, Wang et al^61^ revealed that the level of circ_001988 in CRC was significantly associated with neural infiltration. Circ_103809 levels were notably decreased in the CRC tissues. Zhang et al^62^ found that the expression of circ_103809 was associated with lymph node metastasis, while the downregulated expression of circ_104700 was associated with distant metastasis. There is no doubt that circRNAs play a vital role in tumour metastasis and invasion. Therefore, they could serve as biological markers in gastrointestinal tumours and other neoplastic diseases, and potentially aid in clinical diagnosis and treatment in the future.

### 3.3 CircRNA and the chemoradiotherapy resistance of gastrointestinal tumours

Chemoradiotherapy resistance is mediated by the interaction between internal and external mechanisms. The internal factors include EMT, oxidative regulatory factors, protective autophagy and metabolic plasticity among others. While the external factors include tumour microenvironment, hypoxia and so on. Both internal and external factors act on cancer cells through corresponding pathways, thus reducing their sensitivity to chemoradiotherapy and strengthening their resistance.^63^ The mechanism of chemoradiotherapy resistance has not been fully elucidated thus far, and chemoradiotherapy resistance remains one of the key reasons for the failure of tumour treatment.

There are few reports on the relationship between circRNAs, gastrointestinal tumours and chemoradiotherapy resistance. Li et al found ^64^ that the expression levels of circRNA CBL.11 increased significantly following radiotherapy in CRC, and negatively impacted cancer progression by sponging miR‐6778‐5p and regulating the levels of YWHAE. Therefore, they suggested that circRNA CBL.11 had important implications for the radiotherapy of tumours. Xiong et al ^65^ identified 71 differentially expressed circRNAs resistant to fluorouracil (5‐FU) radiotherapy in CRC cells by microarray analysis, including circ_0000504, circ_0007006, circ_0000237 and circ_0074930. Among these circRNAs, 47 were markedly upregulated and 24 were significantly downregulated, which has provided a direction for follow‐up in the clinical treatment of CRC. Besides, Wang et al ^66^ discovered that the expression level of circ_0001313 was significantly upregulated in CRC cells that were resistant to chemoradiotherapy, while that of miR‐338‐3p, its potential binding molecule, was significantly downregulated. Through circ_0001313 knockout experiments, they also observed that the viability and colony formation ability of the CRC cells decreased following radiotherapy, and in contrast, the activity of caspase‐3 increased. These results suggest that the radiosensitivity of colon cancer cells can potentially be increased by the downregulation of circ_0001313 expression. Furthermore, Huang et al ^67^ found that circRNA AKT3 was overexpressed in GC cell lines that were resistant to cisplatin (CDDP) than in the sensitive lines. By inhibiting miR‐198 and upregulating PIK3R1 gene expression, circRNA AKT3 promoted DNA damage and repair, inhibited apoptosis of GC cells, and increased their resistance to CDDP in vitro and in vivo. In a study exploring the correlation between diosbulbin‐B (DB) and circRNA, it was reported that high dose of DB inhibited cell proliferation and caused cell death, while low dose of DB (12.5 M) had little effect on cell viability. Knockdown of circRNA CDR1 caused GC cell apoptosis even with low doses of DB. Further, the study also revealed that circRNA CDR1 could cause REGγ overexpression in GC cells through sponging miR‐7‐5p. Li et al ^68^ speculated that circRNA CDR1 induces the cytotoxic effect of low dose DB in GC cells by modulating the miR‐7‐5p/REG axis. Recent studies have reported that circRNAs are also involved in chemoradiotherapy resistance in other tumours such as cervical and breast cancer. However, the exact regulatory networks of circRNAs remain unclear, and further studies are needed to elucidate them in the future.

### 3.4 CircRNA and the inflammatory responses of gastrointestinal tumours

In the recent years, the relationship between inflammation and tumour has become the focus of intense research in tumour immunity. It has been suggested that approximately 25% of human tumours are caused by uncontrolled inflammation.^69^, ^70^ Unresolved inflammation can promote the progression of the "inflammation to cancer" response chain and eventually lead to the formation of tumours. These tumours are also known as "nonresolving inflammation‐associated tumours".^71^, ^72^ Increasing evidence suggests that circRNAs influence tumour inflammatory response by influencing inflammatory cells, cytokines, chemokines and other important factors associated with uncontrolled inflammation.

After examining the levels of circRNA in patients with gastritis, GC, GC cell lines and normal gastric tissues, Xie et al^58^ found that the expression level of circ_0074362 was significantly reduced in the first three groups, suggesting that circ_0074362 may affect the transformation of gastritis to GC. Analysis of related circRNAs in colitis‐induced colon cancer mice showed that differentially expressed circRNAs may be involved in inflammation‐induced cancer and the underlying pathogenesis of these circRNAs may involve the mmu‐circ‐001226/mmu‐circ‐000287‐miRNA‐mRNA regulatory network.^73^ Ulcerative colitis (UC) is thought to be closely associated with the development of colon cancer. While exploring the changes in circRNAs and their roles in UC, Wang et al ^74^ ascertained that the expression levels of circ _0007919 in UC continued decrease and was associated with the clinicopathological features and epithelial integrity. This study also suggested that circ _0007919 may be involved in the course of UC by interacting with miR‐138 and let‐7a to regulate the potential target genes, EPC1 and VIPR1. While evaluating the possibility of using circRNAs in monocytes as biological markers of inflammatory bowel disease, Ye et al^75^ found that circ_103516 was associated with the occurrence and development of UC, and may be involved in a series of pathological processes of UC by regulating miR‐19b‐1‐5p. Further, Hsiao et al^76^ discovered that circCCDC66 expression levels were significantly increased in colonic polyps compared with adjacent normal intestinal mucosal tissues. This finding suggests that circRNAs are associated with the pathological process of inflammatory polyps. Overall, there are limited studies on the relationship between circRNAs and inflammation and the development of inflammation‐related diseases. However, with increasing number of studies focusing in these areas, the precise relationship between circRNAs and inflammation and related tumours, and the mechanism through which circRNAs regulate inflammation and promote the development of inflammation towards tumours will eventually become clear.

## 4 ASSOCIATION BETWEEN CIRCRNA AND THE CLINICOPATHOLOGY OF GASTROINTESTINAL TUMOURS

In the recent years, several studies have contributed to a better understanding of circRNAs and their important role in the development of tumours. Studies have shown that circRNAs are not only involved in tumour proliferation, invasion, metastasis, radiochemotherapy resistance and inflammatory response, but are also closely associated with the clinical characteristics of the patients (Table 2).

**Table 2:** The correlation between circRNAs and clinicopathological features in gastrointestinal

	CircRNA	Sample sources	Dyregulation	Relationship with clinicopathology	References
Oesophageal cancer	circ_0006168 circ_100876 circ‐DLG1 circ_0067934 circ_0004370 circ‐SMAD7 circ‑TTC17	Tissue Tissue Tissue, Plasma Tissue Tissue Tissue, Plasma Tissue, Plasma	Up Up Up Up Up Up Up	Lymph node metastasis and TNM stage Invasion depth, lymph node metastasis and vascular infiltration TNM stage Low differentiation, I‐II T stage and I‐II TNM stage Tumour size Lymph node metastasis and TNM stage Lymph node metastasis and TNM stage	^4^ ^136^ ^39^ ^14^ ^77^ ^78^ ^79^
Gastric cancer	circ_0006848 circ HECTD1 circ LMTK2 circ‐DONSON circ PSMC3 circ‐SFMBT2 circ‐KIAA1244 circPVRL3 circ_0000745 circLARP4 circ_0065149 circ_006100 circ_102958 circ_0001821 circ_0074362 circ_0067582 circ_0005758 circ‐EIF4G3 circ‐ERBB2 circ_0081143 circ_0009910 circ_0000467 circ_0066444 circ_0000520 circ_0001649 circ‐ZFR circ_0067582 circ_0005556	Tissue, Plasma Tissue Tissue Tissue Tissue Tissue, Plasma plasma Tissue Tissue, Plasma Tissue Tissue Tissue Tissue Tissue, Plasma Tissue Tissue Tissue Tissue Tissue Tissue Tissue Tissue, Plasma Tissue Tissue, Plasma Tissue Tissue Tissue Tissue	Down Up Down Up Down Up Down Down Down Down Down Up Up Down Down Down Down Up Up Up Up Up Up Down Down Down Down Down	Low differentiation and size of tumour Lymph node metastasis and American Joint Committee on Cancer stage Lymph node metastasis and TNM stage Lymph node metastasis and TNM stage Lymph node metastasis and TNM stage TNM stage Lymph node metastasis and TNM stage TNM stage Tumour differentiation(tissue), TNM staging (plasma) Pathological stage Tumour diameter and nerve infiltration TNM stage, cell differentiation and lymph node metastasis TNM stage Invasion depth, lymph node metastasis Lymph node metastasis CEA level and gastric cancer stage CEA level and perineural infiltration TNM stage and lymph node metastasis Tumour size and invasion depth TNM and lymph node metastasis Distant metastasis, clinical stage and differentiation TNM stage Lymph node metastasis TNM stage (tissue)，CEA level (plasma) Pathological differentiation Related to stage and lymph node metastasis Tumour diameter and carbohydrate antigen 19‐9 Differentiation, TNM stage and lymphatic metastasis	^84^ ^80^ ^81^ ^59^ ^45^ ^43^ ^82^ ^85^ ^87^ ^86^ ^137^ ^104^ ^83^ ^138^ ^58^ ^139^ ^139^ ^140^ ^141^ ^113^ ^142^ ^143^ ^144^ ^145^ ^146^ ^47^ ^147^ ^148^
Colorectal cancer	Circ‐HIPK3 circ_0026344 circ_0000567 circ_0000069 circ_001988 circ_103809 circ_104700 circ‐DDX17 circ_0003906 circ_0007534 circ_0000218 circ_0021977 circ_0004585 circ_0007142 circRNA_104916 circ VAPA circ_0005075 circ‐0104631 circ_0002138 circ_0014717 circ_0142527	Tissue Tissue Tissue Tissue Tissue Tissue Tissue Tissue Tissue Tissue Tissue Tissue Tissue Tissue Tissue Tissue Tissue Tissue Tissue Tissue Tissue	Up Down Down Up Down Down Down Down Down Up Up Down Up Up Down Up Up Up Down Down Down	Lymph node metastasis, distant metastasis and TNM stage Lymph node metastasis Tumour size, lymph node and distant metastasis, TNM stage Age and TNM stage Tumour differentiation and perineural infiltration Lymph node metastasis and TNM stage Distant metastasis Invasion depth, lymph node and distant metastasis, TNM stage Lymph node metastasis and poor differentiation Tumour stage and lymph node metastasis T stage and regional lymph node metastasis TNM stage Tumour size Low differentiation and lymph node metastasis Tumour size, T stage and lymph node metastasis Invasion depth, lymph node and distant metastasis, TNM stage Distant metastasis, invasion depth, TNM stage, tumour diameter TNM stage and distant metastasis Age TNM stage and distant metastasis Age, differentiation invasion, distal metastasis, TNM stage, and carcinoembryonic antigen	^52^ ^93^ ^60^ ^90^ ^61^ ^62^ ^62^ ^92^ ^91^ ^38^ ^124^ ^125^ ^149^ ^126^ ^150^ ^128^ ^151^ ^152^ ^153^ ^154^ ^155^

In the process of exploring the relationship between the clinical and pathological characteristics of OC patients and the expression level of circRNAs, studies have found that the expression levels of circRNAs are related to the size of the tumour. For example, circ_0004370 accelerates the development of OC through miR‐1294/LASP1 pathway, and the expression level of circ_0004370 correlated with the size of OC tissues.^77^ The expression of circRNAs also correlated with lymph node metastasis and TNM staging of the tumours, such as for circ_0006168.^4^ The expression of circ‐SMAD7, which was significantly upregulated in the tissues and serum of patients with OC compared to normal oesophageal tissues and serum, was highly negatively correlated with TNM stage and lymph node metastasis of oesophageal cell carcinoma.^78^ Similarly, Wang et al ^79^ found a significant correlation between circ‐TTC17 and TNM staging and lymph node metastasis in OC. In addition, the expression level of circRNAs is also related to other clinicopathological features. For example, Xia et al^14^ found that the expression level of circRNA 0067934 was related to the poor differentiation of tumours and indicated the T and TNM stages I‐II.

Some studies have found that circRNAs are related to the clinicopathological characteristics of GC, including TNM stage, lymph node metastasis, degree of differentiation and size. For example, circHECTD1, circ‐LMTK2 (which is encoded by the LMTK2 gene) and circ‐KAA124 were reported to be closely related to lymph node metastasis and TNM stage.^80^, ^81^, ^82^ Similarly, Wu et al^42^ found that circ‐DCAF6, which was upregulated in GC, correlated with the invasion depth, lymph node invasion and TNM staging. When studying whether circRNA_102958 could be used as a marker for early GC, Wei et al ^83^ found a positive correlation between its expression level and TNM staging. Circ_0006848, which was related to the ribosomal protein L6, has been reported to be negatively related to tumour hypodifferentiation and size.^84^ In addition, some circRNAs were shown to be related to the survival rate and prognosis of GC patients. For example, Rong et al^45^ confirmed that in GC patients, the expression level of circ‐PSMC3 was closely related to the high stage of TNM and low survival rate. Sun et al^85^ found that in GC, circPVRL3 was negatively correlated with TNM stage and positively correlated with overall survival rate. Compared with normal tissues, downregulated expression of circRNA‐LARP4 in GC patients has been reported to be correlated with pathological staging and poor prognosis.^86^ Besides, the expression level of circRNAs is correlated with different pathological features in different tissues. For example, the expression level of circ_0000745 in GC tissues is related to the differentiation of GC, while in GC serum it is related to TNM staging.^87^

In the context of CRC, based on the expression level of circRNAs in normal and cancer tissues, we divided them into upregulated and downregulated circRNAs. In the upregulated circRNAs, circRNA ciRS‐7‐A was confirmed to be related to the low survival rate of patients.^88^ Jin et al^89^ found that circ_0136666 played an important role in the progression of CRC through the miR‐136/SH2B1 axis, and its expression level in cancer tissues and cell lines correlated with overall survival rate. The expression level of circRNAs is also related to the age of CRC patients and the metastasis of cancer tissues. For example, Guo et al^90^ reported that the expression of circRNA_0000069 was associated with the age of CRC patients and the TNM stage of the tumour. While circHIPK3, which was significantly upregulated in CRC tissues and cell lines, positively correlated with CRC metastasis and advanced clinical symptoms.^52^ Among the circRNAs whose expression is downregulated in CRC compared with normal tissues, circ‐0000567 was related to tumour size, lymph node metastasis, distant metastasis,and TNM stage.^60^ Zhang et al^62^ revealed that circ_103809 and circ_104700 were related to lymph node metastasis and distant metastasis, respectively, in CRC. Similarly, circRNA_0003906 was also associated with lymph node metastasis and poor differentiation.^91^ In addition, some circRNAs were found to be associated with neural infiltration, lymphatic invasion and CRC tissue size. For example, the downregulation of circ‐001988 was involved in the differentiation of CRC and nerve infiltration.^61^ circDDX17 was related to a variety of biological processes in CRC, including lymphatic infiltration, invasion depth, lymph node metastasis, distant metastasis and TNM staging.^92^ In addition, Yuan et al^93^ found that circ_0026344 was not only associated with the progression of CRC, but also with lymph node metastasis. Zhang et al^21^ demonstrated that differential expression of circ_0000826 was closely associated with tumour size, TNM staging and distant metastasis in CRC patients.

Taken together, these studies showed that circRNAs have a variety of important biological effects in gastrointestinal neoplasms. Among the biological functions, the most important and useful function is its potential in early diagnosis and therapeutics. The stable differential expression of some circRNAs in the serum of tumour patients can be used as early diagnostic markers. In addition, studies on the mechanism of circRNAs can provide more effective clinical solutions for the treatment of gastrointestinal tumours.

## 5 DISCUSSION AND PROSPECTS

As a newly emerging class of RNA molecules, circRNAs were initially considered to be functionless by‐products of aberrant splicing. However, with the development of high‐throughput sequencing and related technologies in recent decades, more and more studies have shown that circRNAs play a vital role in oncology and many other fields. For example, circRNAs can regulate the translation of functional proteins, act as ceRNAs to influence the transcription process and so on, making them potential targets in the treatment of gastrointestinal tumours. In recent years, circRNAs have gained more attention as a potential new drug target. circRNAs can be packed into exosomes or other nanostructured materials, and play a role by being transported to the corresponding target cells through tumour microenvironment, which may provide the potential targets for the therapy of tumours.^94^ However, at present, the research on circRNAs as a therapeutic target of gastrointestinal tumours is still in the stage of animal trial. Ju et al^95^ demonstrated that the ability of lung metastasis was significantly reduced in mice injected with hsa_circ_0079480 knockdown cells compared with those injected with control colon cancer cells. In the therapy of other diseases, circRNAs can also show great advantages as a therapeutic target. For example, circAnks1a can mediate the physiological process of neuropathic pain by promoting the expression of VEGFB in the mice model of spinal nerve ligation.^96^ However, a long path needs to be traversed from animal experiment to clinical application. A key challenge for circRNAs as a therapeutic drug target is how to take the inhibitors or agonist of circRNAs into the specific target cells and ensure their stable expression in the target cells.

In addition, because of their covalently closed circular structure, circRNAs are not easily degraded by nucleases. Therefore, they can be used as important biomarkers in the serum and tissues for the diagnosis of gastrointestinal tumours.^29^, ^97^ CircRNAs can also be used as a diagnostic kit in clinical research. The fluorescence in situ hybridization (FISH) kit of circRNAs has the advantages of safety, rapidity, high sensitivity and simultaneous display of multiple colours, which can be used to detect the abnormal expression of circRNAs in the progression of tumours. Because circRNAs are such a new class of molecules, our understanding of their functions is still rather limited in many respects; 1) The process of formation of circRNAs may not be limited to what has been described in studies thus far, and its origin and mode of formation remain to be explored further; 2) Although high‐throughput sequencing has shown that many circRNAs are abnormally expressed in tumour tissues, the specific mechanisms and functions of circRNAs have not been fully understood; 3) Previous studies have shown that many circRNAs can regulate gene transcription and translation through sponging miRNAs, but the specific target axis may not be limited to one or two axes; 4) Some circRNAs have the ability to translate proteins, but the role of the translated proteins in tumour development is still unclear, and the translation function of most circRNAs have not been confirmed; 5) A growing number of studies have focused on whether circRNAs can be used as biomarkers for the diagnosis and treatment of certain tumours, but few clinical trials have confirmed their feasibility. Therefore, further research is in needed to explore the relationship between circRNAs and gastrointestinal tumours.

## 6 CONCLUSIONS

This review mainly discussed the role of circRNAs in the development and clinicopathological characteristics of digestive tract tumours. We briefly highlighted the connecting links among circRNAs, miRNAs and target genes, and summarized the biological roles of circRNAs in the three major digestive tract tumours. Finally, we discussed relationship between circRNAs expression and clinicopathological characteristics, all of which will lay a solid foundation for further functional studies of various circRNAs.

## CONFLICTS OF INTEREST

The authors declare no competing financial interest.

## **AUTHOR** **CONTRIBUTION**

HN and YT.W. involved in writing—original draft preparation. HN YT.W. ZM.L. CL.O. and JH.Z involved in writing—review and editing. HN YT.W. and CL.O. involved in visualization. CL.O. and JH.Z. involved in supervision, project administration and funding acquisition.

## Data Availability

The data that support the findings of this study are available from the corresponding author upon reasonable request.
